# Association Between ALK Rearrangement and Ultra-Late Recurrence in Lung Cancer: Case Report and Pooled Analysis

**DOI:** 10.7759/cureus.51354

**Published:** 2023-12-30

**Authors:** Jamila Mammadova, Tawee Tanvetyanon

**Affiliations:** 1 Department of Hematology and Oncology, University of South Florida, Tampa, USA; 2 Department of Thoracic Oncology, Moffitt Cancer Center and Research Institute, Tampa, USA

**Keywords:** non-small cell lung cancer (nsclc), ultra-late recurrence, recurrence, alk rearrangement, lung cancer

## Abstract

Ultra-late recurrence, defined as recurrence occurring 10 years or longer after curative treatment, is uncommon for non-small cell lung cancer (NSCLC). To date, factors associated with ultra-late recurrence remain unknown. We report a case with ultra-late recurrence and reviewed the literature published during 2010-2023.

This is a case of a 66-year-old woman, with a significant smoking history and a previous history of lung adenocarcinoma, who underwent surgery for a brain metastasis detected on imaging. The pathology confirmed lung adenocarcinoma with an epidermal growth factor receptor (EGFR) exon 20 insertion, a finding consistent with the initial lung surgery a decade ago. With receiving intrathecal topotecan, the patient has maintained stable disease 10 months post-surgery.

Given the rarity of ultra-late recurrence of NSCLC, we also conducted a pooled analysis with the outcome of interest being a time to recurrence. Data from this case report was analyzed along with previously published 26 cases of ultra-late recurrence. Multivariable analysis indicated that the only factor significantly predicting time to recurrence was anaplastic lymphoma kinase (ALK) rearrangement.

## Introduction

Following curative surgery, patients with early-stage non-small cell lung cancer (NSCLC) still face a risk of recurrent disease. This risk typically increases along with the cancer stage. For example, in a study of 1619 patients with stage I to III NSCLC, recurrences occurred in 22% of stage I patients, 33% among stage II patients, and 40% among stage III patients [1]. Most recurrences occurred within the first two years after surgery and higher stages predicted earlier recurrence. For instance, in stage II and III lung cancers, 80% of all recurrences occurred within two years, compared with only 60% among stage I. However, regardless of stage, almost all recurrences can be expected to have occurred by five years after surgery. Currently, the national guideline recommends surveillance imaging for the first five years after definitive treatment followed by optional yearly surveillance thereafter [2].

Late recurrence, defined as beyond five years after surgery as well as ultra-late recurrence, defined as beyond 10 years after surgery, have been rarely investigated in NSCLC. In a study of 517 patients with stage IA NSCLC, there were 22 patients (4.8%) who could be considered as having a late recurrence, with the greatest time to recurrence being 113 months [3]. Ultra-late recurrence is even less commonly reported. In a study from Japan containing 1458 resected NSCLC patients, 12 patients (2.5%) developed recurrence later than 10 years after surgery [4]. A study of late and ultra-late recurrence NSCLC can be challenging due to the needed prolonged follow-up time as well as the difficulty in differentiating between recurrent and new primary lung cancer. The criteria proposed by Martini and Melamed which are based on histological similarity, mediastinal lymph node involvement, and a two-year disease-free interval, can be helpful [5]. In addition, genetic alteration can also be used to confirm that both initial and recurrent cancers share the same clonal origin.

To date, there is still a paucity of literature on ultra-late recurrence in NSCLC. The topic of ultra-late recurrence is clinically relevant because it may have an implication on the duration and extent of surveillance follow-up. In this article, we report on a patient who developed ultra-late recurrence, presenting as brain metastasis after over 10 years from resection. The tissue specimens from both the initial resection of stage IA lung cancer and the resection of brain metastasis were compared. The same clonality was confirmed based on the presence of epidermal growth factor receptor (EGFR) exon 20 insertion in both specimens. We also performed a literature review and conducted a pooled analysis to identify factors associated with time to recurrence among NSCLC cases developing ultra-late recurrence.

## Case presentation

**Figure 1:**
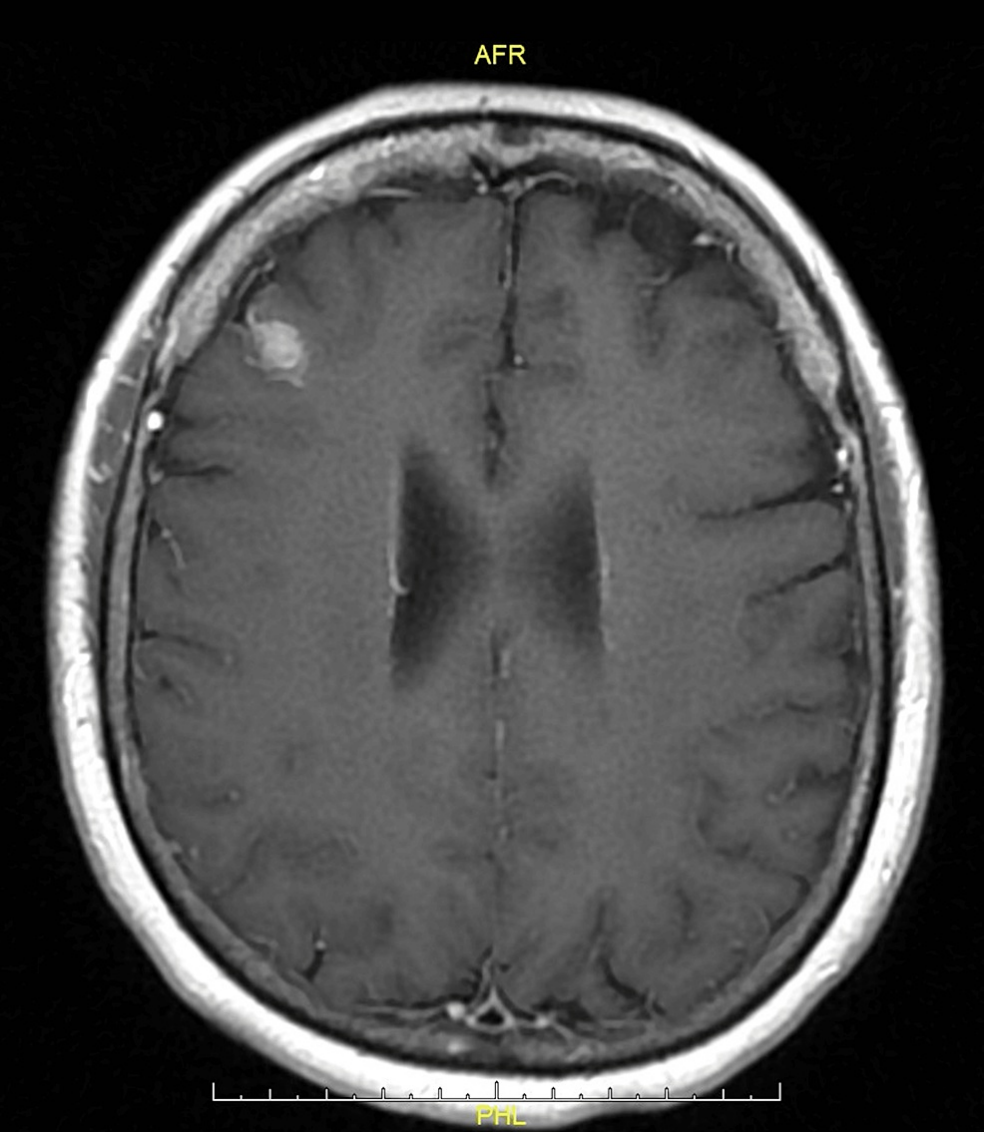
Magnetic resonance imaging T1 axial image of brain

A 66-year-old female patient with a past medical history of cervical cancer, breast cancer, and a 30-pack-year smoking history presented with progressive headaches. Over 10 years ago, a left lower lobectomy was performed for stage T1bN0M0, IA2, well-differentiated adenocarcinoma. Originally, the mass in the left lower lobe was detected during a surveillance computer tomography (CT) scan indicated for the patient's 30-year history of smoking. The patient received no adjuvant chemotherapy. At presentation, a magnetic resonance imaging (MRI) of the brain revealed a solitary 1 cm lesion in the right frontal lobe as well as an enhancement suggesting leptomeningeal involvement (Figure 1). Cerebrospinal fluid examination revealed atypical cells highly suspicious for malignancy. Computerized and positron emission tomography demonstrated no extracranial disease.

The repeated head MRI with and without contrast demonstrated metastatic disease with new parenchymal and leptomeningeal deposits. The patient underwent right frontal stereotactic craniotomy with tractography under stereotactic guidance and Ommaya insertion, followed by whole-brain radiation. Pathological examination showed adenocarcinoma of the lung, positive for thyroid transcription factor 1 and CK7 and negative for CK20, GATA3, mammaglobin, CDX2, and mutant BRAF-V600E. Mismatch repair protein expression was retained (MLH1, PMS2, MSH2, MSH6). A targeted next-generation sequencing analysis revealed an in-frame EGFR exon 20 insertion. We retrieved the specimen from initial lung surgery 10 years ago for comparison. The histology was similar, and EGFR exon 20 insertion was also identified.

At the time of this report, 10 months after surgery, the patient continued to receive intrathecal topotecan with stable disease. 

## Discussion

Literature review and pooled analysis

We performed a literature search on MEDLINE Pubmed and Google Scholar published from January 2010 to 2023 for late or ultra-late recurrence in NSCLC. The reference list of each retrieved article was checked for additional publications. Ultra-late recurrence was defined as the time between the initial surgery and the first recurrence of ≥10 years. Abstracts and full reports were reviewed to extract patient and cancer characteristics including EGFR or anaplastic lymphoma kinase (ALK) alterations. For uniformity of analysis, tumor sizes and stages were converted into the American Joint Committee on Cancer Staging 8th edition [6], when feasible. A pooled analysis was conducted with the outcome of interest being time to recurrence defined as time from initial surgery to the ultra-late recurrence. Descriptive statistics, including median and range, were used for continuous variables as well as percentage and count, for categorical variables. Non-parametric Mann-Whitney U-test was used to compare age. Pearson Chi-square or Fisher exact test was used to compare categorical variables. Median time to recurrence was estimated by the method of Kaplan-Meier and the log-rank test was used to compare groups. Multivariable analysis was performed using Cox Proportional Hazard models. Variable selection was performed using backward stepwise elimination method with a significant level to stay set at 0.10. All p-values were 2-tailed and significance level was set at <0.05. Analyses and graphics were performed on SPSS version 28 (IBM, Armonk, NY, USA). 

Results

Publication and Patient Characteristics

Our literature search identified 16 publications describing 27 cases with ultra-late recurrence, including the case described in this report (Table 1). Most publications emanated from institutions in Japan, contributing to 21 patients. The remaining publications were from institutions in Canada, Italy, Denmark, Korea, and the United States. The largest report was published in 2019, containing 12 cases.

**Table 1:** Reported cases of ultra-late recurrence of lung cancer in literature NR = not reported; EFGR = epidermal growth factor receptor; ALK = anaplastic lymphoma kinase

Patient number	First author, year [reference]	Sex	Age (years)	Time to recurrence (years)	Smoking history	Initial stage	Biomarker status
1	Inaoka, 2010 [7]	M	51	15.0	NR	NR	NR
2	Murakami, 2010 [8]	F	40	20.0	No	T1bN1	ALK
3	Noroxe, 2012 [9]	M	50	10.0	Yes	T2aN1	Wild type
4	Tomizawa, 2013 [10]	M	43	15.0	No	T2aN0	ALK
5	Tsukamoto, 2014 [11]	M	43	15.0	No	T1cN2	ALK
6	Al-Bhimani, 2015 [12]	M	41	17.0	Yes	T1N0	ALK
7	Ishida, 2015 [13]	M	58	19.0	NR	T2aN0	ALK
8	Matsumoto, 2016 [14]	M	58	22.0	Yes	T3N2	ALK
9	Zito Marino, 2016 [15]	M	35	10.0	No	T2aN0	ALK
10	Sonoda, 2017 [16]	M	61	19.0	NR	T2aN0	Wild type
11	Isono, 2018 [17]	M	45	11.0	Yes	T1aN1	ALK
12	Sonoda, 2019 [4]	F	61	10.1	No	T1aN1	Wild type
13	Sonoda, 2019 [4]	F	70	10.1	No	T1bN1	Wild type
14	Sonoda, 2019 [4]	M	70	10.3	Yes	T1bN0	Wild type
15	Sonoda, 2019 [4]	F	70	10.4	No	T1aN0	EGFR exon 19
16	Sonoda, 2019 [4]	F	49	11.4	Yes	T2aN0	Wild type
17	Sonoda, 2019 [4]	M	58	11.6	Yes	T1bN0	EGFR exon 21
18	Sonoda, 2019 [4]	F	56	12.6	No	T1aN1	EGFR exon 21
19	Sonoda, 2019 [4]	F	69	13.0	No	T1cN1	EGFR exon 20
20	Sonoda, 2019 [4]	M	65	13.4	Yes	T3N0	Wild type
21	Sonoda, 2019 [4]	M	50	13.8	Yes	T2aN1	NR
22	Sonoda, 2019 [4]	F	49	17.8	No	T2aN0	ALK
23	Sonoda, 2019 [4]	M	55	19.8	Yes	T3N0	ALK
24	Matsuo, 2020 [18]	F	54	16.0	No	T1cN2	ALK
25	Yang, 2021 [19]	M	30	11.7	NR	T2aN0	KRAS G12D
26	Park, 2023 [20]	F	56	10.5	No	T3N1	RET KIF13A
27	Current case	F	66	10.3	Yes	T1bN0	EGFR exon 20

Patient characteristics were obtained from 27 patients (Table 2). There were five patients with EGFR mutation, 11 patients with ALK rearrangement, and 11 patients with no EGFR or ALK alterations or unknown biomarker status. Among those without EGFR or ALK alterations, one patient had a KRAS mutation, and one patient had a RET rearrangement. Overall, the median age of patients was 54 years (range 30-70 years). All of those with molecular alterations had both specimens from resection and specimens from recurrence examined to confirm biomarker similarity except for patient number 2 due to the degradation of initial tissue and patient number 9 due to the inadequate biopsy sample in the recurrence cancer. Patient number 9 did achieve a partial tumor response to ALK inhibitor treatment to further substantiate the presence of ALK rearrangement.

**Table 2:** Characteristic of patients and initial tumor by EGFR/ALK status Values for characteristics are median (IQR) or N (%). A p-value <0.05 was considered statistically significant. EFGR = epidermal growth factor receptor; ALK = anaplastic lymphoma kinase; IQR = interquartile range

Characteristics	ALK-rearranged patients	EGFR-mutated patients	No EGFR or ALK alteration	Total patients	p-value
	(N=11)	(N=5)	(N=11)	(N=27)	
Median age (range)	45.0 (35-58)	58.0 (56-70)	51.0 (30-70)	54.0 (30-70)	0.03
Histology:					
-Adenocarcinoma	11 (100)	5 (100)	10 (91)	26 (96)	0.47
-Atypical carcinoid	0 (0)	0 (0)	1 (9)	1 (4)	
Sex:					
-Female	3 (27)	4 (80)	4 (36)	11 (41)	0.13
-Male	8 (73)	1 (20)	7 (63)	16 (59)	
Smoking history:					
-No	6 (60)	3 (60)	3 (38)	12 (52)	0.59
-Yes	4 (40)	2 (40)	5 (63)	11 (48)	
Nodal involvement:					
-No	7 (64)	4 (80)	5 (50)	16 (62)	0.52
-Yes	4 (36)	1 (20)	5 (50)	10 (38)	
T stage:					
-T1	5 (45)	5 (100)	3 (30)	13 (50)	0.04
-T2-T3	6 (55)	0 (0)	7 (70)	13 (50)	
N stage:					
-N0	7 (64)	4 (80)	5 (50)	16 (62)	0.02
-N1	0 (0)	1 (20)	5 (50)	6 (23)	
-N2	4 (36)	0 (0)	0 (0)	4 (15)	
Stage group:					
-Stage I	6 (55)	4 (80)	4 (40)	14 (54)	0.11
-Stage II	1 (9)	1 (20)	5 (50)	7 (27)	
-Stage III	4 (36)	0 (0)	1 (10)	5 (19)	

Patients with ALK rearrangement were younger than those of patients with EGFR mutation or with no ALK/EGFR alteration: 49 years compared with 58 and 61 years, respectively (p=0.001). The oldest patient with ALK rearrangement was 58 years while the oldest patient in this series was 70 years. No significant difference was found in terms of sex, smoking history, or nodal involvement. There was a statistically higher proportion of patients with N-2 disease among those with ALK rearrangement.

Ultra-Late Recurrence

**Figure 2:**
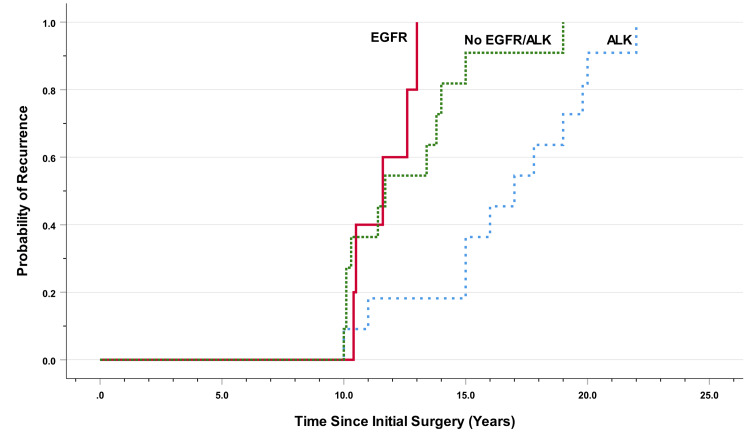
Time to recurrence by EGFR/ALK status EFGR = epidermal growth factor receptor; ALK = anaplastic lymphoma kinase

Time to recurrence was analyzed among 27 patients. The median time to recurrence was 13.4 years (95% CI: 11.4-15.4 months). Patients with ALK rearrangement had a significantly longer time to recurrence than other groups of patients, with a median of 17.0 years (95% CI: 13.9-20.0) compared with 11.6 years (95% CI: 9.3-13.9) among those with EGFR mutation and 11.7 years (95% CI: 8.4-15.0) among those without EGFR or ALK alteration, p=0.004 (Figure 2). 

We performed univariable and multivariable analyses considering biomarkers, sex, age, smoking status, nodal involvement, T stage, and stage group (Table 3). Only ALK rearrangement was found to be an independent predictor of delayed time to recurrence, with a hazard ratio of 0.30 (95% CI: 0.13-0.73, p=0.008). Finally, we reviewed the pattern of recurrence among patients with ALK rearrangement (Table 4). In all patients, the recurrence could be visualized by routine computerized tomography of the chest including the upper abdomen.

**Table 3:** Factors associated with time to recurrence Values are univariable hazard ratios (95% CI). A p-value <0.05 was considered statistically significant. CI = confidence interval; EFGR = epidermal growth factor receptor; ALK = anaplastic lymphoma kinase

Factors	Univariable Hazard Ratio (95% CI)	p-value
Age:		
-Per year increase	1.01 (0.97-1.06)	0.58
Sex:		
-Female	1.41 (0.64-3.09)	0.39
-Male	Reference	
Smoking history:		
-Yes	0.93 (0.40-2.18)	0.88
-No	Reference	
T-stage:		
-T1	1.75 (0.78-3.90)	0.18
-T2 or T3	Reference	
Node positive:		
-Yes	1.19 (0.52-2.73)	0.68
-No	Reference	
Gene alteration:		
-ALK rearrangement	0.30 (0.13-0.73)	0.008
-EGFR mutation, none, unknown	Reference	
Stage group:		
-Per stage increase	0.87 (0.53-1.43)	0.59

**Table 4:** ALK testing methodology and pattern of recurrence *Patient developed recurrence in the ipsilateral hilar lymph node. ALK = anaplastic lymphoma kinase; FISH = fluorescence in situ hybridization using ALK break-apart probe; IHC = immunohistochemistry; NA = not available; RT-PCR = reverse transcription polymerase chain reaction followed by direct sequencing using antisense strands.

Patient number	Test in initial cancer	Test in recurrent cancer	Presence of pulmonary metastasis	Presence of lesion at surgical site	Presence of extra-thoracic metastasis	Sites of distant metastasis at recurrence
2	NA	RT-PCR, FISH, IHC	Yes	No	No	Pleura, bilateral lungs
4	RT-PCR, FISH	RT-PCR, FISH	No	Yes	No	None
5	RT-PCR, FISH, IHC	RT-PCR, IHC	Yes	No	Yes	Contralateral lung, abdominal lymph node
6	FISH, IHC	FISH, IHC	No	No	No	Pleura
7	FISH, IHC	FISH, IHC	Yes	No	No	Bilateral lungs
8	IHC	FISH	No	No	No	Chest wall, sternum
9	FISH, IHC	NA	No	Yes	No	None
11	IHC	IHC	No	Yes	Yes	Pleura, mediastinal and supraclavicular node
22	IHC	IHC	Yes	No	No	Ipsilateral lung
23*	IHC	IHC	No	No	No	None
24	RT-PCR, IHC	RT-PCR, IHC	No	No	Yes	Brain, liver

Discussion

In this article, we reported a case of ultra-late recurrence adenocarcinoma with EGFR exon 20 insertion. To our knowledge, only four previous cases of ultra-late recurrence with EGFR mutation have been reported in the literature. Nonetheless, our literature review showed that there are many more cases of ultra-late recurrence with ALK rearrangement. A pooled analysis of reported cases demonstrated that ALK rearrangement was associated with a longer time to recurrence than EGFR mutation or no ALK/EGFR alterations.

To our knowledge, the current report contains the largest number of patients with ultra-late recurrence and is the first to explore potential factors associated with the phenomenon. In pulmonary adenocarcinoma, EGFR mutation is generally more common than ALK rearrangement. Specifically, while EGFR mutation is present ranging from 15% in Western countries to 50% in Asian countries, ALK rearrangement is present in only 5-10% of patients [21]. On the contrary, we found that among NSCLC with ultra-late recurrence, there were more cases of ALK rearrangement than EGFR mutation. This finding may be explained, in part, by the fact that patients with ALK rearrangement are generally younger than those with EGFR mutation, thus longer life expectancy to allow for a longer observation period for recurrence. However, age alone was unlikely to be the single explanation, given a much larger pool of patients without ALK rearrangement and the fact that age was not found to be a significant predictor of time to recurrence in this small study.

Very little research has been conducted for ultra-late recurrence in NSCLC; however, in breast cancer, ultra-late recurrence has been associated with tumors with positive estrogen receptor (ER) status. The recurrence risk of ER-positive breast cancer following surgery is initially lower than ER-negative disease; however, it will surpass the risk among ER-negative cancer after about eight years after surgery and will persist for up to 25 years [22]. Some authors have proposed that late recurrence is the result of metastatic cancer becoming dormant and later being reactivated as patients age [23]. However, it remains unknown what causes cancer cells to become dormant. There is no evidence to suggest that EGFR or ALK-positive adenocarcinomas of the lung have a slower growth rate compared to other lung cancers. In previous studies of resected early-stage NSCLC, recurrence during five to 10 years after surgery has been associated with factors such as race, histology, and vascular invasion [1,3]. Unfortunately, no information on EGFR or ALK was available.

Our study suggests a unique biological characteristic of ALK-rearranged lung cancer in its association with ultra-late recurrence. Nonetheless, some limitations should be noted. As this work is based largely on published studies, it can suffer from reporting and publication bias. However, the bias should be equally present for both EGFR and ALK cancers, thus minimizing its impact on the statistical comparison. Another consideration is that most reported cases are from Asian centers, and this may limit its generalizability.

## Conclusions

In summary, among NSCLC cases with ultra-late recurrence, ALK rearrangement is prevalent and ALK rearrangement is associated with greater time to recurrence than EGFR mutation. Given the potential implication for long-term follow-up need among patients with ALK rearrangement, it may be advisable to check resected early-stage lung cancer for ALK rearrangement especially among younger patients.

## References

[1] Karacz CM, Yan J, Zhu H, Gerber DE (2020). Timing, sites, and correlates of lung cancer recurrence. Clin Lung Cancer.

[2] Ettinger DS, Wood DE, Aisner DL (2023). NCCN Guidelines® Insights: non-small cell lung cancer, version 2.2023. J Natl Compr Canc Netw.

[3] Maeda R, Yoshida J, Ishii G (2010). Long-term outcome and late recurrence in patients with completely resected stage IA non-small cell lung cancer. J Thorac Oncol.

[4] Sonoda D, Matsuura Y, Ichinose J (2019). Ultra-late recurrence of non-small cell lung cancer over 10 years after curative resection. Cancer Manag Res.

[5] Martini N, Bains MS, Burt ME (1995). Incidence of local recurrence and second primary tumors in resected stage I lung cancer. J Thorac Cardiovasc Surg.

[6] Chansky K, Detterbeck FC, Nicholson AG (2017). The IASLC lung cancer staging project: external validation of the revision of the TNM stage groupings in the eighth edition of the TNM classification of lung cancer. J Thorac Oncol.

[7] Inaoka T, Takahashi K, Aburano T, Miyokawa N, Tandai S, Kobayashi T, Matsuno T (2010). Spinal metastasis from lung cancer fifteen years after surgery presenting a pseudohemangioma appearance of the vertebra: a case report. Spine (Phila Pa 1976).

[8] Murakami S, Yokose T, Saito H (2010). Recurrent EML4-ALK-associated lung adenocarcinoma with a slow clinical course. Lung Cancer.

[9] Nørøxe DS, Sørensen JB (2012). Ultra-late relapse with a single cerebellar metastasis 10 years after complete surgery for stage IIA non-small cell lung cancer (bronchioalveolar carcinoma). J Thorac Oncol.

[10] Tomizawa K, Ito S, Suda K (2013). Solitary pulmonary metastasis from lung cancer harboring EML4-ALK after a 15-year disease-free interval. Lung Cancer.

[11] Tsukamoto Y, Kanamori K, Watanabe T (2014). Recurrence of lung adenocarcinoma after an interval of 15 years revealed by demonstration of the same type of EML4-ALK fusion gene. Pathol Res Pract.

[12] Al-Baimani K, Sekhon HS, Wheatley-Price P (2015). Recurrence of anaplastic lymphoma kinase (ALK) positive adenocarcinoma after 17 years: case report. Cancer Treat Commu.

[13] Ishida J, Suzuki H, Kitamura Y, Uchida T (2015). [Recurrence of anaplastic lymphoma kinase positive lung cancer nineteen-year after the primary surgery]. Kyobu Geka.

[14] Matsumoto D, Takizawa H, Takashima M (2016). Chest wall metastasis of anaplastic lymphoma kinase-positive lung adenocarcinoma 22 years after lobectomy. Jpn J Lung Cancer.

[15] Zito Marino F, Morabito A, Gridelli C (2016). Crizotinib response in a late relapse of ALK-positive lung adenocarcinoma. Appl Immunohistochem Mol Morphol.

[16] Sonoda D, Mikubo M, Shiomi K, Satoh Y (2017). Complete resection of oligorecurrence of stage I lung adenocarcinoma 19 years after operation. Ann Thorac Surg.

[17] Isono T, Yuasa M, Tani M (2018). Postoperative recurrence of anaplastic lymphoma kinase (ALK)-positive lung adenocarcinoma after 11 years. Jpn J Lung Cancer.

[18] Matsuo S, Tsukamoto Y, Mabuchi E (2020). A case of late distant recurrence/metastasis (≧10 years after curative surgery) of anaplastic lymphoma kinase-rearranged lung cancer and the review of similar cases in the literature. Hum Pathol Case Rep.

[19] Yang SR, Chang JC, Leduc C (2021). Invasive mucinous adenocarcinomas with spatially separate lung lesions: analysis of clonal relationship by comparative molecular profiling. J Thorac Oncol.

[20] Park HY, Park JH, Shin MG (2023). Case report: a case of ultra-late recurrence of KIF13A-RET fusion non-small cell lung cancer response to selpercatinib. Front Oncol.

[21] Costa PA, Saul EE, Paul Y, Iyer S, da Silva LL, Tamariz L, Lopes G (2021). Prevalence of targetable mutations in black patients with lung cancer: a systematic review and meta-analysis. JCO Oncol Pract.

[22] Colleoni M, Sun Z, Price KN (2016). Annual hazard rates of recurrence for breast cancer during 24 years of follow-up: results from the international breast cancer study group trials I to V. J Clin Oncol.

[23] Narod SA, Giannakeas V, Sopik V (2020). Late recurrences after estrogen receptor-positive breast cancer. JAMA Oncol.

